# Structure-Based Design of Small-Molecule Inhibitors of Human Interleukin-6

**DOI:** 10.3390/molecules30142919

**Published:** 2025-07-10

**Authors:** Ankit Joshi, Zhousheng Xiao, Shreya Suman, Connor Cooper, Khanh Ha, James A. Carson, Leigh Darryl Quarles, Jeremy C. Smith, Madhulika Gupta

**Affiliations:** 1 Computational Biophysics Lab, Indian Institute of Technology (Indian School of Mines) Dhanbad, Dhanbad 826004, Jharkhand, India; 2 Division of Nephrology, University of Tennessee Health Science Center, Memphis, TN 38163, USA; 3 Biosciences Division, Oak Ridge National Lab, Oak Ridge, TN 37831, USA; 4 Tickle College of Engineering, University of Tennessee, Knoxville, TN 37996, USA; 5 Integrative Muscle Biology Laboratory, Division of Regenerative and Rehabilitation Sciences, College of Health Professions, University of Tennessee Health Science Center, Memphis, TN 38163, USA

**Keywords:** Human Interleukin-6, molecular dynamics, ensemble docking, binding affinity, inhibitors, small molecule antagonist

## Abstract

Human Interleukin-6 (hIL-6) is a pro inflammatory cytokine that binds to its receptor, IL-6Rα followed by binding to gp130 and subsequent dimerization to form a hexamer signaling complex. As a critical inflammation mediator, hIL-6 is associated with a diverse range of diseases and monoclonal antibodies in clinical use that either target IL-6Rα or hIL-6 to inhibit signaling. Here, we perform high-throughput structure-based computational screening using ensemble docking for small-molecule antagonists for which the target conformations were taken from 600 ns long molecular dynamics simulations of the apo protein. Prior knowledge of the contact sites from binary complex studies and experimental work was incorporated into the docking studies. The top 20 scoring ligands from the in silico studies after post analysis were subjected to in vitro functional assays. Among these compounds, the ligand with the second-highest calculated binding affinity experimentally showed an ~84% inhibitory effect on IL6-induced STAT3 reporter activity at 10 μM concentration. This finding may pave the way for designing small-molecule inhibitors of hIL-6 of therapeutic significance.

## 1. Introduction 

Human interleukin-6 (hIL-6) is a multifunctional cytokine, the release of which is triggered by leukocytes, adipocytes, fibroblasts, endothelial cells, keratinocytes, and other cytokines such as IL-1 and tumor necrosis factor (TNF) as an immunologic response to infections and tissue injuries. hIL-6 transmits signals within cells by binding to either its specific receptor, IL-6Rα, or a natural soluble form of this receptor, sIL-6Rα, to form a binary complex that binds to the ubiquitously expressed glycoprotein coreceptor gp130, also referred to as IL-6R [1,2]. Signal transduction occurs when the ternary complex IL-6/IL-6Rα/gp130 dimerizes to form a hexamer comprising two molecules each of IL-6, IL-6Rα, and gp130. This signaling complex activates Janus kinases and tyrosine kinases (JAK1, JAK2, TYK2), as well as signal transducers and activators of transcription (STAT), in both the JAK/STAT and RAS/MAPK pathways [3,4]. 

The binding of hIL-6 to IL-6Rα is referred to as ‘classical’ signaling and is responsible for the role of hIL-6 in host defense mechanisms and the stimulation of hepatocytes to produce acute-phase proteins [5]. In contrast, the binding of hIL-6 to sIL6-Rα is referred to as ‘trans’ signaling and is accountable for the immediate inflammatory reactions that occur in chronic conditions such as rheumatoid arthritis, Crohn’s disease, Castleman disease, and inflammatory bowel disease [5]. Trans signaling also mediates tumor microenvironments leading to malignancies such as multiple myeloma, colon, and pancreatic cancers [6,7]. The IL-6/sIL-6R complex can disrupt bone equilibrium by stimulating the formation of osteoclasts, resulting in abnormal bone loss, heightened fragility, and the development of arthritic conditions [8]. IL-6 in combination with TNF-α facilitates the calcification of vascular cells to induce persistent low-grade systemic inflammation in chronic kidney disease (CKD) and progression of exacerbated diabetic nephropathy (DN) [9]. Patients with different neoplastic disorders have been found to have elevated concentrations of IL-6, associated with a higher risk of cardiovascular mortality [9,10]. Recent research has also highlighted the significance of hIL-6 in severe instances of COVID-19, where it is a vital mediator of the cytokine storm syndrome that can lead to adult respiratory distress syndrome (ARDS) [11]. Thus, hIL-6 has been implicated in the pathophysiology of multiple diseases, and this makes it an important therapeutic target. 

Investigative approaches for therapeutic development so far have involved either reducing the formation of hIL-6 or inhibiting the binding of hIL-6 to IL-6Rα by targeting hIL-6 or the receptor. One of the popular antibodies on the market, Tocilizumab, targets IL-6Rα to treat rheumatoid arthritis, juvenile idiopathic arthritis, and Castleman disease [12]. This drug demonstrates superior effectiveness compared to methotrexate in the treatment of juvenile idiopathic arthritis and reduces hepcidin levels [13,14]. Tocilizumab has demonstrated efficacy in patients with rheumatoid arthritis who did not respond to TNF antagonist therapy [15]. However, attempts to repurpose Tocilizumab for multiple myeloma and kidney transplantation showed inconsistencies in their results and raised safety concerns [16]. Sarilumab is an approved drug for rheumatoid arthritis and Castleman disease that targets IL-6Rα [17], while Vobarilizumab is undergoing phase 3 clinical trials for rheumatoid arthritis [12]. Siltuximab, an approved hIL-6 antibody for treating multicentric Castleman disease, has been observed to decrease iron-related complications in patients [18,19]. Further, Ancrile et al. [20] showed that the growth of Ras-driven tumors could be suppressed by targeting hIL-6 using specific antibodies. Olamkicept selectively targets trans signaling for inflammatory bowel disease and is undergoing phase 2 trials [21]. Another inhibitor of the complex is the sgp130Fc protein, which is formed by fusing two sgp130 molecules to human IgG1-Fc [21,22,23] based on the fact that the sgp130 protein functions as an intriguing natural antagonist of the hIL-6/sIL-6Rα complex [24]. However, an excess of sgp130 may adversely interfere with the classical hIL-6 signaling [25,26]. Thus, the pleiotropy of hIL-6 makes it particularly challenging to develop specific inhibitors that selectively target the pro inflammatory pathway of hIL-6.

Small-molecule JAK1 inhibitors such as tofacitinib and baricitinib have been approved for rheumatoid arthritis but lack specificity and pose an increased risk of viral respiratory tract infection [27]. Other small-molecule JAK1 inhibitors, such as fligotinib and upadacitinib, are in phase 3 clinical trials for rheumatoid arthritis and inflammatory bowel disease [12,28,29,30]. LMT-28 is a novel synthetic IL-6 inhibitor that suppresses phosphorylation of STAT3, gp130, and JAK2 and reduces IL-6-dependent TF-1 cell proliferation in mice models [31]. In addition, several other small-molecule candidates have emerged as potential IL-6 inhibitors. However, none of these are specific to IL-6. IDC-24, a 1,3-indanedione derivative identified via in silico design, has demonstrated superior binding affinity and stability compared to LMT-28 [32]. Methotrexate, though traditionally used as an immunosuppressant, has also been proposed to modulate IL-6 activity based on docking studies. Additionally, carbohydrate–small molecule hybrids such as LS-TG-2P and LS-TF-3P have shown promising inhibition of gp130-mediated IL-6 signaling [33]. Other notable compounds include pyrrolidinesulphonylaryl derivatives like compound 6a, which selectively inhibit IL-6/STAT3 signaling [34], and natural product-based leads such as Lead5, which interfere with gp130 dimerization and downstream activation [35]. Despite the involvement of hIL-6 in a plethora of diseases, the available drugs on the market that directly interact with the protein are limited to antibodies that treat rheumatoid arthritis and Castleman disease. It may be speculated that the role of hIL-6 in these two conditions is well-defined as increased concentration levels of hIL-6 are directly linked to the progression of the disease [15,36]. 

It is evident that the general challenges involved in the development of small-molecule inhibitors for hIL6 that target specific diseases are multifold. Different diseases involving hIL6 show differences in the pathology and the associated symptoms that need to be studied and characterized clearly. The situation is further complicated by the variations in phenotype of the affected individuals who may show varied responses to the same drug with side effects ranging from mild to severe [37,38]. Since the biologics in clinical use are confined to monoclonal antibodies that target hIL-6 or IL-6Rα, the development of economically viable small-molecule inhibitors with the added advantage of ease of administration can be a boon to treating diseases involving hIL-6. Clearly, there is an unmet need to develop orally administered small molecule hIL-6 inhibitors, and we postulate that this may be achievable using ‘rational’ structure-based drug design. The research efforts directed towards developmental strategies for such potential lead drug molecules can be an asset in targeting the multiple diseases involving hIL-6 and improving upon existing drugs, with the possibility of enhanced efficacy and safety profiles.

Experimental high-throughput screening (HTS) is an expensive method for finding active small-molecules that also suffer from hit rates well below 0.1% [39]. A more effective and economical approach is to use structure-based computations in combination with assays of selected molecules. This approach to developing small compounds to target hIL-6 leverages an understanding of the interactions that drive the formation of the key complex, hIL-6/IL-6Rα, in the hIL-6 signaling cascade. The binding of hIL-6 to IL-6Rα is of paramount importance in the signaling pathway of hIL-6, as gp130 molecules cannot directly bind to hIL-6 in the absence of IL-6Rα. Thus, contact sites on hIL-6, as elucidated from hIL-6/IL-6Rα binding studies, can be targeted to identify potential lead compounds. Our previous study using extensive molecular dynamics simulations revealed that the binding of hIL-6 to IL-6Rα reduces the rigidity of residues 48 to 58 in the flexible AB loop region due to the disruption of hydrogen bonds [40]. In contrast, residues 59-78 of the AB loop region lose their plasticity and become more rigid by forming contacts with the receptor on binding. Thus, the binding of hIL-6 to IL-6Rα induces structural and dynamic changes in the AB loop region of hIL-6 that subsequently facilitate the formation of hIL-6/IL-6Rα for further binding to gp130. In addition, the residues comprising helix D in hIL-6 are involved in stabilizing the interactions with the IL-6Rα primarily through two salt bridge interactions. Thus, the residues present in both the AB loop region and helix D facilitate the formation of the hIL-6/IL-6Rα complex via an interplay of electrostatic, hydrophobic, hydrogen bonding, and aromatic stacking interactions. 

Previous studies have used pharmacophore modeling, docking, and structure–activity relationships to identify suitable hits for hIL-6 [41,42,43]. However, a comprehensive analysis of the changes induced in hIL-6 on binding with the receptor using molecular dynamics simulations and selective targeting of specific binding sites to design small molecule antagonists has not been done yet. Moreover, the docking studies to date on hIL-6 have been performed using the crystal structure, and the docking algorithms may not sample all the different conformational states that the protein can have, which play a crucial role in antigen–antibody recognition involving hIL-6 [41,42,43,44]. In this work, we use the conformations sampled from the molecular dynamics (MD) simulations of apo hIL-6 of Ref. [37] to perform ensemble docking on various database sets to identify potential antagonists of hIL-6. After performing several drug likeness checks and scrutinizing for PAINS, the top twenty scoring compounds from in silico studies were subjected to an in vitro functional assay. The ligand with the second highest binding affinity from docking calculations was observed to show ~84% inhibition of hIL-6 and thus represents a lead compound for further development. 

Traditional HTS methods involve the brute-force screening of thousands to millions of compounds against a single static protein conformation, often failing to account for target flexibility, particularly for proteins like cytokines that engage in dynamic protein–protein interactions (PPIs). In contrast, our study employs a rational structure-based design approach, incorporating ensemble docking based on conformational clusters extracted from a 600 ns MD simulation of apo hIL-6. This allows for dynamic sampling of binding-competent states and accounts for receptor flexibility that is often missed in static HTS protocols. This strategy is especially valuable for proteins like IL-6, where interaction surfaces are shallow and transient. By leveraging MD-derived conformational ensembles, our docking protocol targets biologically relevant and structurally diverse receptor states. This significantly enhances the likelihood of identifying ligands that bind to functional epitopes, such as the AB loop and helix D, which are critical to receptor complex formation. Ensemble docking has been shown to outperform conventional single/crystal structure docking for targets with high conformational plasticity [44,45,46,47]. Moreover, unlike Siltuximab, a monoclonal antibody that binds IL-6 and prevents receptor binding through steric hindrance [48], our approach is focused on discovering small molecules that modulate the same interaction by directly targeting the cytokine, potentially offering improved pharmacokinetics, oral bioavailability, and manufacturing scalability.

## 2. Materials and Methods

**Figure 1:**
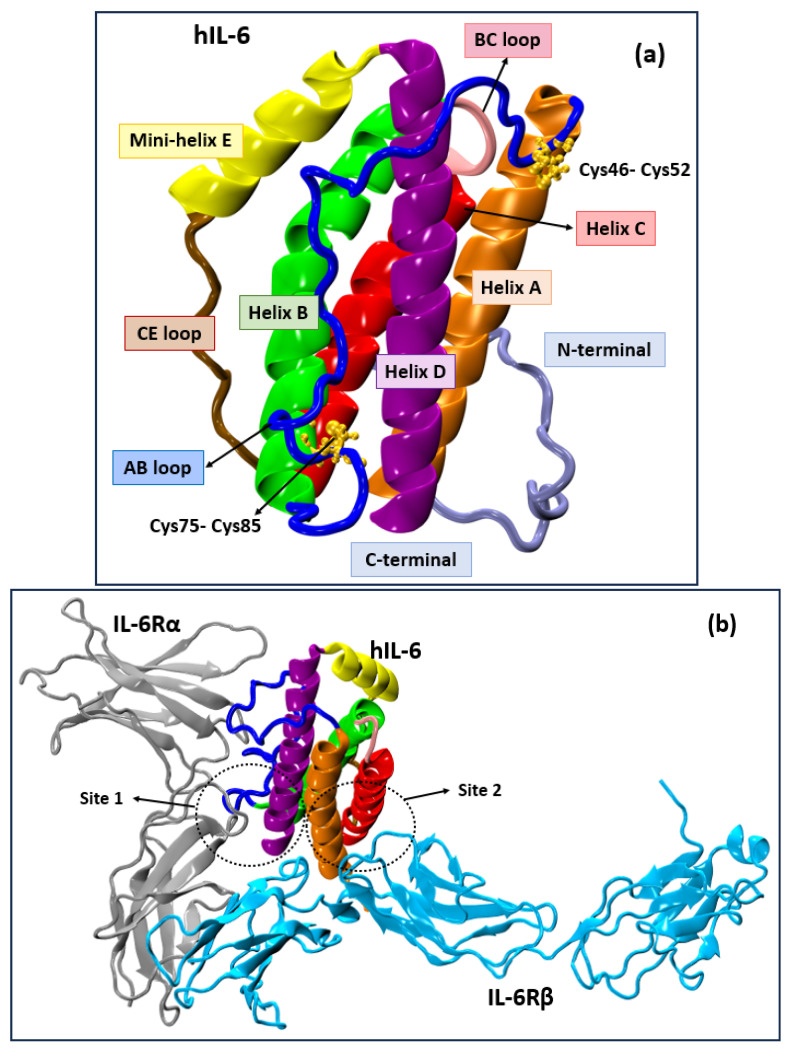
(**a**) Structure of hIL-6; (**b**) crystal structure of hIL-6/IL-6Rα/gp130 complex (PDB id:1P9M). The helices A, B, C, and D of hIL-6 are shown in orange, green, red, and purple, respectively. The CD loop comprises mini-helix E (yellow) and the CE loop (brown), while the blue and pink depict loops AB and BC of hIL-6.

hIL-6 has 186 amino acid residues and a distinct secondary and tertiary structure shown in Figure 1a. The protein consists of four α-helices: A (Ser23-Cys46), B (Glu82-Phe107), C (Glu111 to Ala132), and D (Gln158-Met186) with three interhelical loops, AB (Glu41-Asn81), BC (residues 108-110), and CD (Lys133-Ala155). The CD loop is made up of a CE loop (Lys133-Asp142) and a short mini-helix E (Asp142-Ala155). The helices A and B are oriented in the same direction while helices C and D are opposite. The two pairs of disulfide bonds formed by Cys46 (helix A)-Cys52 (AB loop) and Cys75 (AB loop)-Cys85 (helix B) stabilize the interhelix connection in hIL-6. The sites involved in binding of hIL-6 to IL-6Rα and gp130 are shown in Figure 1b. As proteins are highly flexible molecules, it is useful to take their flexibility into account while performing docking studies. Thus, instead of docking to only a crystal structure, we consider different conformations of the protein and the ligand, in ensemble docking [45].

### 2.1. Homology Modeling

PDB IDs 1ALU (apo hIL-6) and 1P9M (hIL-6/hIL-6Rα/gp130 complex) [49,50] were used to perform homology modeling using ROSETTA (2018.09) [51] to simulate the absent residues in the crystal structure of hIL-6. Both, 1ALU and 1P9M lacked clear electron density for the 18 *N*-terminal residues. 1ALU has higher resolution than 1P9M but had 8 residues missing in the AB loop region compared to 3 residues for ALU. Consequently, a weight of 1 and 0 was assigned to 1ALU and 1P9M, respectively, during the construction of the structures. The highest-scoring model was utilized to initiate molecular dynamics simulations.

### 2.2. Molecular Dynamics (MD) Simulations

The hIL-6 modeled structure was solvated with TIP3P water [52] and neutralized with one K^+^ using CHARMM-GUI [53]. All-atom MD simulations were carried out using a GPU-accelerated version of NAMD 2.13 with the CHARMM36 force field [54,55].

The conjugate gradient method was used to perform minimization of the system followed by heating up to 300 K in the constant temperature and pressure ensemble (NPT). The equilibration and production run with total run length of 600 ns was performed in the canonical NVT ensemble. The Langevin dynamics method [56], with a damping coefficient of 1ps^−1^ was used to maintain the temperature. A Nose-Hoover barostat [56] with an oscillation period of 1ps^−1^ was used to maintain the pressure at 1 atm. A cut-off of 12 Å was used for van-der Waals forces, with a switch distance of 10 Å. The electrostatic interactions were computed using the Particle Mesh Ewald (PME) [57] algorithm, with a grid spacing of 1 Å. The SHAKE algorithm [58] was used to restrict the lengths of all bonds involving hydrogen atoms. All simulations were performed using an integration time step of 2 fs. The configurations in the production runs were dumped at intervals of 2 ps. Three sets of independent molecular dynamics simulations, each for 600 ns, were performed for apo hIL-6 (cumulative 1800 ns). The reported values of root mean square deviations and root mean square fluctuations showed less than 1% error over the three simulations using the standard error formula [40]. Thus, the run length of 600 ns was adequate to sample the conformational space of hIL-6, and one of the simulations was used in the next section to generate the different ensembles for docking.

### 2.3. Ensemble Docking Algorithm to Select Distinct Conformations of HIL-6 for Screening

Ensemble docking studies were performed here using MD simulations of apo hIL-6 for 600 ns [40]. The KFC2 server [59,60] was used to calculate hotspots for the hIL6/IL-6Ra complex to identify key contact sites for binding in the crystal structure (1P9M) as well as in our simulations. The results reveal that Phe76, Gln77, Gln177, Leu180, Arg181, Ala182 and Arg184 of hIL-6 were the predicted hotspots. These data are in excellent agreement with experimental studies suggesting the role of residues 178-186 of hIL-6 in protein-receptor binding [61,62,63,64]. This information was incorporated into our clustering protocol to obtain the conformationally different structures. We used the root mean square deviations (RMSDs) of residues within 5 Å of residues 178–186 to cluster MD-derived protein structures using the GROMOS method in GROMACS [65] with a cutoff of 2 Å. Using RMSD = 2.5 Å resulted in only 3 different conformations of hIL-6 that may be too low to represent the different conformational states of hIL-6. It may be noted here that the cut-off value for clustering should be chosen to obtain an optimal number of different conformations of the protein. A high cut-off value for RMSD would give few conformations, leading to inadequate representation of the different conformations possible for the protein. A low cutoff value would result in too many conformations of the protein that may not be too different from each other to yield different binding energies in docking. A total of eight distinct conformations of hIL-6 were obtained using a cutoff of 2 Å in this work to show a good representation of the different conformations for hIL-6 to account for its flexibility while docking.

### 2.4. Docking Protocol and Selection of Database for Docking

The program VinaMPI, a high-throughput docking version of AutodockVina, was used to speed up the calculations [66] and targeted docking was performed using prior information about the binding site in hIL-6 [40,61,64]. The NCI database (265, 242 compounds) [67], Enamine diversity set (~50,000 compounds) [68], Enamine PPI library (40,640 compounds) [69], and SWEETLEAD database (4030 approved drugs, herbal isolates, and medicines) [70] were used to perform high-throughput screening to identify potential ligands for targeting hIL-6. Thus, a total of ≈360,000 compounds were docked with eight different conformations of hIL-6. OPEN BABEL and MGLTools were used to generate structure files [71,72]. A box of 30 Å × 30 Å × 30 Å centered around the 178–186 residues of hIL-6 was chosen for docking. The exhaustiveness parameter in AutodockVina was set to 50. 

### 2.5. Selection Criteria for Choosing Compounds for In Vitro Screening

The ligands were arranged in the order of their docking scores and were subjected to drug likeness checks. A PAINS filter was also used to screen out compounds with sub-structural features that make them promiscuous compounds in high-throughput screening using FAF-Drugs4 software [73]. In addition, other promiscuous binders identified in the group were also excluded from the list [46,74]. Lipinski’s rule of five, which was introduced in 1997, is commonly referred to as a guideline for identifying drug-like compounds. Its main purpose is to tackle the issue of low solubility and permeability in oral medications. This is achieved by ensuring that the molecular weight (MW) is less than 500, the lipophilicity (cLogP) is less than 5, the number of hydrogen bond donors (OH + NH groups) is less than 5, and the number of hydrogen bond acceptors (O + N atoms) is less than 10 [75,76,77]. The Lipinski rule of five was applied to assess the drug-likeness of the top-scoring ligands [78]. A strict implementation of Lipinski’s rule has been observed to pose a barrier in some cases to exploring novel drugs [79]. Therefore, if fewer than two rules are violated, a non-zero drug-likeness value is assigned to the ligand. The ligands were also checked for the absence of any reactive segments based on such as metals, N/O/S-N/O/S single bonds, thiols, acyl halides, Michael acceptors, azides, esters, and others [80]. Compounds that formed only hydrophobic interactions or had fewer than 2 hydrogen bonds were removed from the list. The top 20 compounds obtained thereafter were subjected to in vitro experimental studies. 

### 2.6. In Vitro Functional Assays

The top 20 scoring compounds were purchased from Enamine (Kyiv, Ukraine). HEK293T cells purchased from ATCC (Manassas, VA, USA) were initially reported to be mycoplasma-free and were cultured in Dulbecco’s modified Eagle’s medium (DMEM) containing 10% fetal bovine serum and 1% penicillin and streptomycin (P/S). We used the Universal Mycoplasma Detection Kit (30–1012 k) from ATCC to detect mycoplasma contaminants in cell culture. The cells were used for experiments until passage 20. For IL6-mediated activation of the IL-6Rα/gp130/STAT3 signaling, 6 × 10^6^ HEK293T cells were transiently transfected with either an empty expression vector (4.0 µg) or full-length human IL-6 (4.0 µg) [81], along with IL-6Rα (4.0 µg) [82], gp130 (4.0 µg) [83], and STAT3 luciferase reporter (2.0 µg) [84] (Addgene, Inc., Watertown, MA, USA), and Renilla luciferase-null (0.6 µg) (Promega, Madison, WI, USA) as an internal control plasmid. Transfections were performed by electroporation with voltage 250 V, capacitance 950 µF, and pulse length 15 ms within 0.4 cm cuvettes using Cell Line Nucleofector Kit R according to the manufacturer’s protocol (Amaxa Inc., Seattle, WA, USA) [85]. The cells were planted in a 12-well plate at a density of 5 × 10^5^ per well. Thirty-six hours after transfection, the cells were treated with the test compound at 10 μM or in a range of 10^−8^–10^−4^ M and the vehicle only served as the control. After 5 h, the cells were lysed by 150 µL lysis buffer, and luciferase activities were measured using a Synergy H4 Hybrid Multi-Mode Microplate Reader and Promega Dual-Luciferase Reporter Assay System (Madison, WI, USA). Firefly luciferase reporter activity was normalized to the Renilla control. The compound-mediated fold inhibition in STAT3 activity over vehicle was calculated. 

Three independent experiments were run for each scenario to obtain sufficient statistics. Statistical significance between two groups was evaluated by the unpaired 2-tailed *t*-test, and that between multiple groups was evaluated by one-way analysis of variance (ANOVA) with the Turkey multiple comparison test. These calculations were performed using GraphPad Prism 5.0 (San Diego, CA, USA). The IC_50_ of Z169667518 was obtained graphically from concentration–effect curves using GraphPad Prism 5.0 (San Diego, CA, USA).

## 3. Results and Discussion

**Figure 2:**
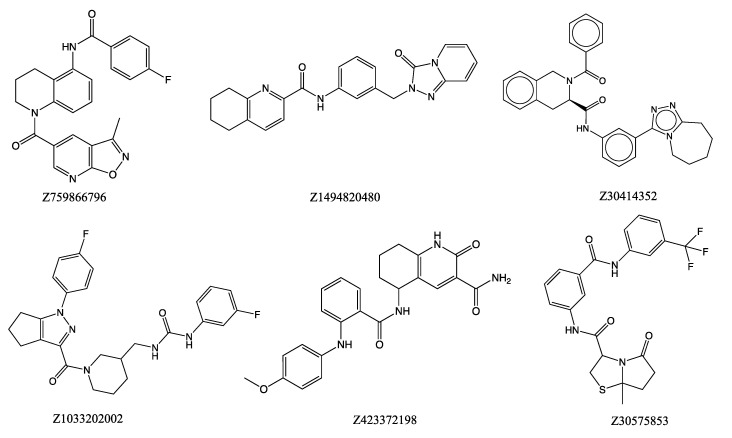

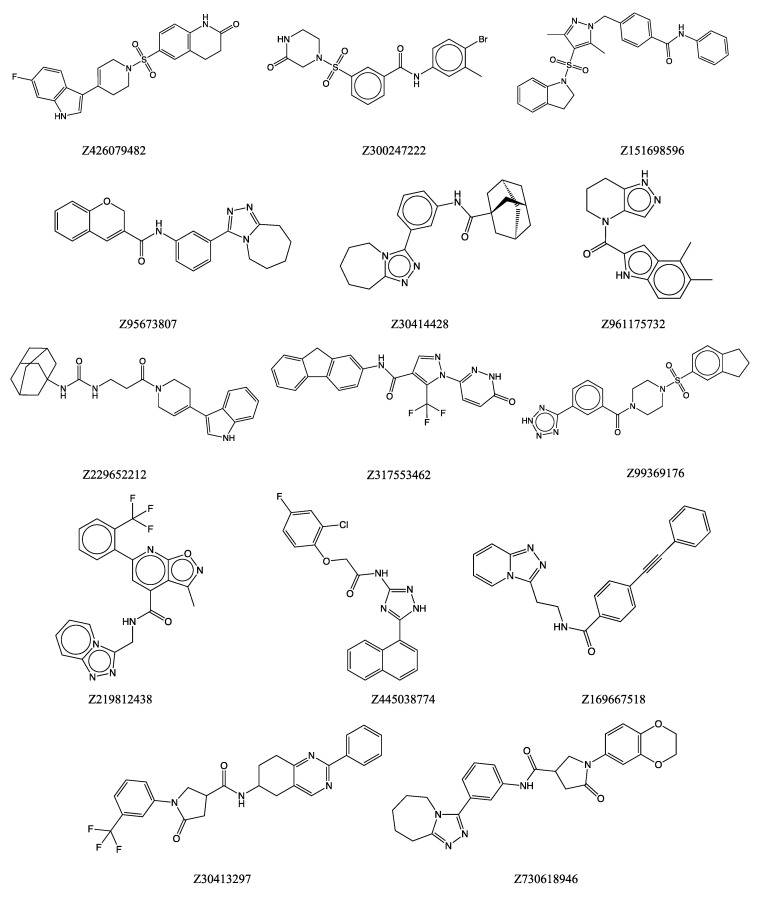
Chemical structures of top 20 scored ligands from Autodock Vina.

In silico studies: High-throughput virtual ensemble docking was performed on the conformations derived from extensive MD simulations with the goal of identifying candidate small molecules that bind to hIL-6 and prevent the formation of the hIL-6/IL-6Rα complex. The chemical structures of the remaining top 20 ligands with the highest binding affinities, as calculated with Autodock Vina, are shown in Figure 2. All 20 ligands are highly hydrophobic and mostly aromatic. Table 1 lists physical properties related to drug likeness of the top docked compounds. The molecular weights of all the reported compounds are less than 500. All 20 compounds have logP < 5 except Z445038774. The other rules relating to the number of hydrogen bond donors and acceptors were also followed for the top docked ligands. Interestingly, all the compounds listed in Table 1 are found to be from the Enamine database. This emphasizes the importance of small, well-curated, targeted libraries in binding proteins in certain specific cases. Moreover, since hIL-6 interacts with the receptor at the interface of the helix rather than having a binding pocket or cavity, it may be speculated that the enamine diversity set can be used for inhibiting protein–protein interactions compared to the NCI and SWEETLEAD databases. Table 1 also shows topological polar surface area, tPSA, which is the key parameter to predict drug permeability and binding affinity. Compounds with high tPSA values (>140 Å^2^) are typically less likely to be orally bioavailable.

**Figure 3:**
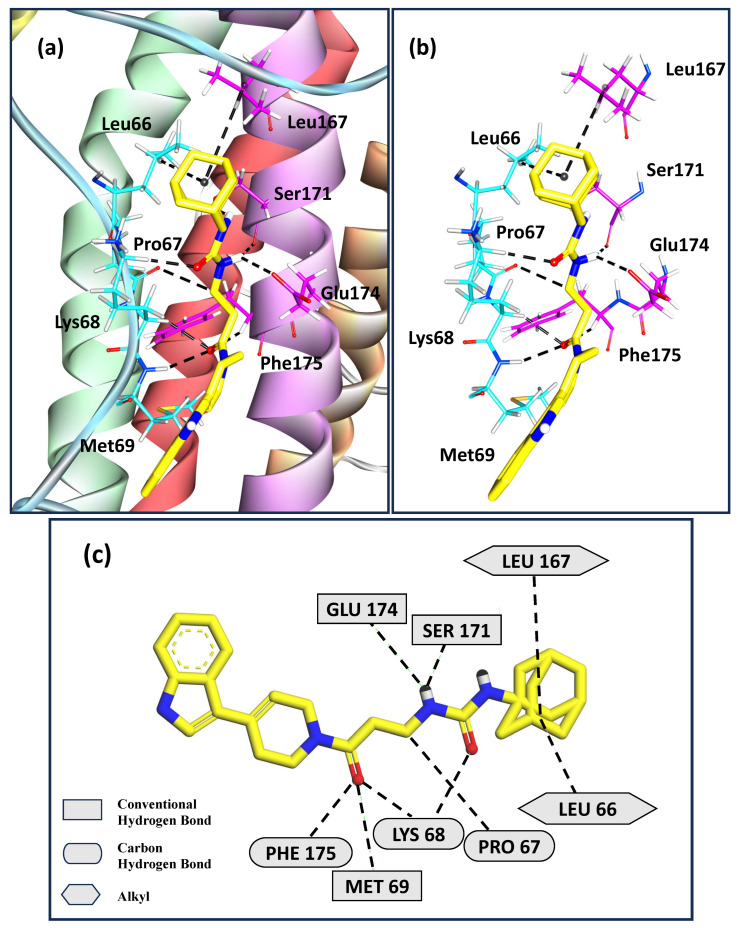
(**a**) Docked pose for the highest binding score ligand Z229652212 with hIL-6; (**b**) and (**c**) represent the sites of interaction of ligand Z229652212 and hIL-6.

Figure 3a depicts the calculated binding pose for the ligand with the highest calculated binding affinity in our docking studies—Z229652212. This ligand interacts with helix D and ABloop region that together comprise site I for binding to hIL-6Rα, as shown in Figure 3b,c. The alkyl groups present in Leu66 and Leu167 interact with the ligand. Met69, Ser171, and Glu174 are involved in forming hydrogen bonds with the ligand. Pro67, Lys68, and Phe175 form carbon-hydrogen bonds with the ligand. This type of non-conventional hydrogen bonding has gained importance recently over a decade and has been observed to play a crucial role in stabilizing the protein–ligand interactions [86].

**Figure 4:**
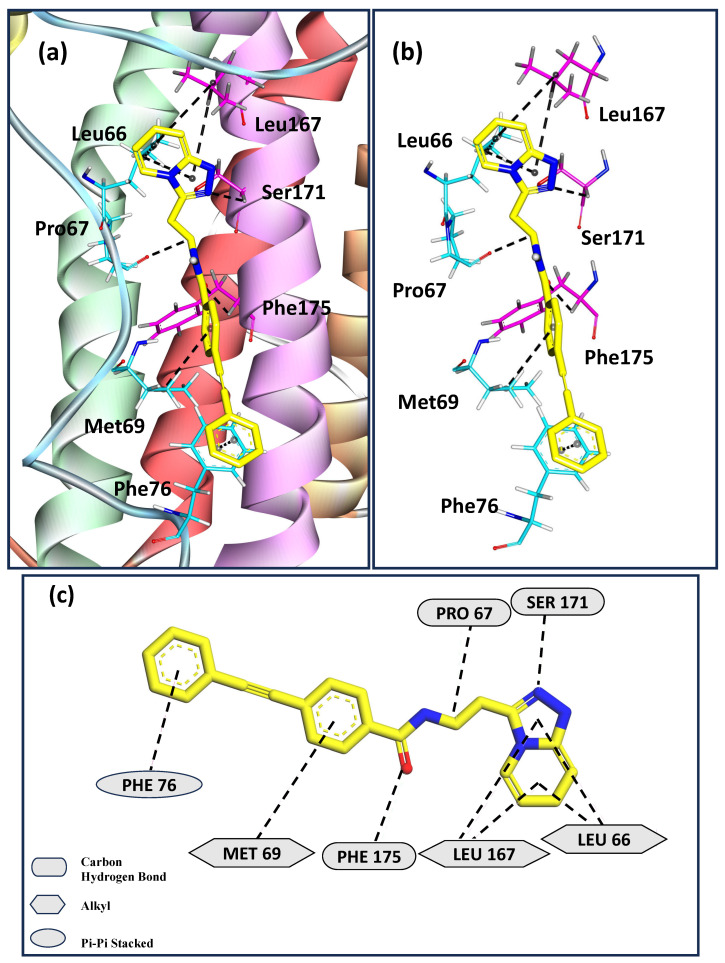
(**a**) Docked pose for the second highest binding score ligand Z169667518 with hIL-6; (**b**) and (**c**) represent the sites of interaction of ligand Z169667518 and hIL-6.

Figure 4a depicts the binding pose for the ligand Z169667518, which shows the second-highest calculated binding affinity with hIL-6. Figure 4b,c show that Leu66, Pro67, Met69, and Phe76 from the AB loop region, with Leu167, Ser171, and Phe175 of helix D of hIL6, interact with the ligand. Leu66, Met69, and Leu167 stabilize the ligand through alkyl interactions. Pro67, Ser171, and Phe175 form carbon–hydrogen bonds with the ligand. Phe76 further stabilizes the protein-ligand complex by π-π stacking interactions. The same residues of hIL-6 involved in stabilizing the interactions with the ligands Z229652212 and Z169667518 in this study were observed to form contacts with the receptor, leading to their rigidification in our previous study [40]. In particular, Phe residues and aromatic rings play a crucial role in stabilizing the hIL6/hIL-6Rα complex. Thus, the results from this work are consistent with our previous study [40] on the role played by the AB loop region in facilitating antigen–antibody recognition in addition to the binding site present on helix D.

**Table 1:** Drug likeness properties [87] of top 20 ligands with the highest docking scores computed from AutoDock Vina ^a^.

Compound ID	Mol. Formula	Mol. Wt.	logP	Net Charge	H-Bond Donors	H-Bond Acceptors	tPSA	Docking Score (kcal/mol)
Z229652212	C_27_H_34_N_4_O_2_	446.595	4.442	0	3	2	77	−9.150
Z169667518	C_23_H_18_N_4_O	366.424	3.102	0	1	4	59	−8.881
Z30414428	C_24_H_30_N_4_O	390.531	4.826	0	1	4	59	−8.840
Z423372198	C_24_H_24_N_4_O_4_	432.48	3.033	0	4	5	126	−8.774
Z30575853	C_22_H_20_F_3_N_3_O_3_S	463.481	4.35	0	2	4	78	−8.711
Z219812438	C_22_H_15_F_3_N_6_O_2_	452.396	4.19	0	1	7	98	−8.701
Z95673807	C_23_H_22_N_4_O_2_	386.455	4.086	0	1	5	69	−8.627
Z1494820480	C_23_H_21_N_5_O_2_	399.454	3.07	0	1	6	81	−8.618
Z30414352	C_30_H_29_N_5_O_2_	491.595	4.877	0	1	5	80	−8.611
Z730618946	C_26_H_23_F_3_N_4_O_2_	480.49	4.189	0	1	4	75	−8.582
Z30413297	C_26_H_27_N_5_O_4_	473.533	3.434	0	1	7	98	−8.574
Z99369176	C_21_H_22_N_6_O_3_S	438.513	1.502	-	1	6	-	−8.287
Z151698596	C_27_H_26_N_4_O_3_S	486.597	4.552	0	1	5	84	−8.281
Z759866796	C_24_H_19_FN_4_O_3_	430.439	4.516	0	1	5	88	−8.258
Z317553462	C_22_H_14_F_3_N_5_O_2_	437.381	4.21	0	2	5	92	−8.238
Z426079482	C_22_H_20_FN_3_O_3_S	425.485	3.67	0	2	3	82	−8.208
Z1033202002	C_26_H_27_F_2_N_5_O_2_	479.531	4.313	0	2	4	79	−7.879
Z961175732	C_17_H_18_N_4_O	294.358	3.101	0	2	2	64	−7.772
Z300247222	C_18_H_18_BrN_3_O_4_S	452.33	2.13	0	2	4	95	−7.331
Z445038774	C_20_H_14_C_l_FN_4_O_2_	396.809	5.084	0	2	4	-	−7.280

^a^ The above table lists the docking ID, molecular formula (Mol. Formula), molecular weight (Mol. Wt.), lipophilicity (logP), net charge, number of hydrogen bond donors, number of hydrogen bond acceptors, topological polar surface area (tPSA) as obtained from Zn database [87] and docking score for each ligand as obtained from AutoDock Vina.

**Figure 5:**
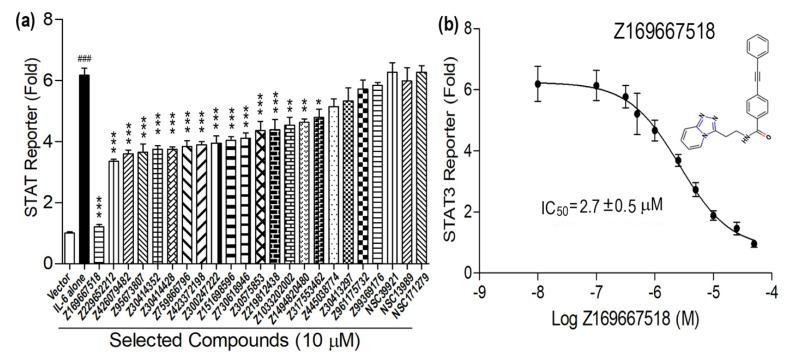
In vitro functional assays of the top 20 compounds from Enamine and 3 from NCI database selected from the in silico virtual screening. (**a**) Effects of these compounds on IL6 induced STAT3 reporter activities in the transiently IL6Rα/GP130-transfected HEK 293T cells. (**b**) Dose−response curve of Z169667518 on IL6-induced STAT3 reporter activities. Data are the means ± SD from three independent experiments. ^###^ Indicates statistically significant difference from empty vector group. *, **, *** indicates statistically significant difference from IL-6 vehicle group at *p* < 0.05, *p* < 0.01, *p* < 0.001, respectively *p* values were determined by one-way ANOVA with Tukey’s multiple-comparisons test.

In vitro screening of the top 20 calculated ligands for antagonization of IL6-mediated activation of IL6Rα/GP130/STAT3 signaling in vitro: 

Using the in vitro screening assay described in Section 2.4, we experimentally tested 20 high-scoring chemical probes identified from the in silico virtual screen at an initial concentration of 10 μM in the presence of IL6, as shown in Figure 5. Sixteen of the twenty compounds exhibited statistically significant measurable effects in inhibiting IL6-stimulated STAT3 reporter activity (Figure 5a). Compounds Z445038774, Z30413297, Z961175732, and Z99369176 at a 10 μM concentration had no significant effect on IL6-induced signal transduction. 

At the 10 μM concentration, 15 compounds exhibited partial (less than 50%) inhibition of IL6-induced STAT3 reporter activity, while one compound, Z169667518, stood out by exhibiting an ~84% inhibitory effect (Figure 5a). In the ensemble docking studies, this compound was found to interact strongly with hIL6, as shown in Table 1 and Figure 4, and had the second-highest calculated binding affinity.

To explore the dose-response effects of Z169667518 we performed additional studies using doses ranging from 10^−8^ to 10^−4^ M. Z169667518 exhibited dose-dependent inhibition of IL6-induced STAT3 reporter activity (Figure 5b). The estimated median inhibitory concentration (IC_50_) value for Z169667518 was 2.7 ± 0.5 µM (Figure 5b). Optimization of this lead compound could potentially result in IL6/IL6Rα/GP130 interaction inhibitors with sub-micromolar to nanomolar binding affinities for IL-6.

**Figure 6:**
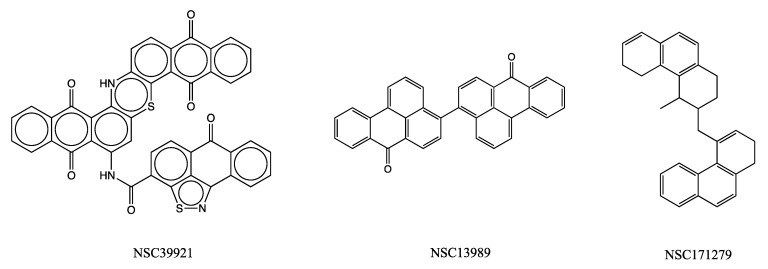
Chemical structures of top 3 scoring NCI compounds subjected to in vitro studies.

It may be noted that despite the elimination of NCI compounds during the filtering criteria discussed in Section 2.5, three of the top-scoring NCI compounds (Figure 6, Table 2) were subjected to in vitro screening. Figure 5 depicts that none of these compounds showed any significant effect on IL6-induced signal transduction, validating their exclusion during the selection criteria as well.

Notably, several studies have been focused on identifying antagonists for hIL-6 using in silico and in vivo methods. However, poor oral bioavailability, difficulty in modulating shallow and flexible protein–protein interaction interfaces, and the lack of structural validation of inhibitor-binding modes have been identified as some of the key bottlenecks associated with successfully identifying small molecule inhibitors for hIL-6 [42,43,88]. These challenges are further compounded by the intrinsic structural plasticity of IL-6, which complicates accurate ligand binding predictions and reduces the reliability of traditional single-conformation docking strategies [43,89]. Additionally, most small molecules reported to date lack a comprehensive experimental characterization, suffer from low selectivity, or fail to engage dynamic binding pockets effectively [88]. Many candidates also do not progress beyond in vitro validation due to pharmacokinetic liabilities such as poor solubility or metabolic instability [32,33]. Thus, the development of effective small molecular inhibitors for hIL-6 by overcoming these limitations is the need of the hour. 

One of the important small-molecule inhibitors, LMT-28 binds to gp130 to limit further proliferation induced by hIL-6. In contrast, the lead compound Z169667518 investigated in this work binds directly to the contact sites on hIL-6 that undergo significant conformational rearrangement during receptor engagement. This novel binding mode potentially enables a more effective disruption of the IL-6/IL-6Rα interaction.

Limitations and Future studies: The present study provides important insights into the identification of potential small-molecule inhibitors for hIL-6. However, several methodological refinements remain to be addressed in future work. While qualitative assessments support the use of the homology model, quantitative structure validation tools such as Ramachandran plot and ERRAT scoring were not employed here. These assessments play an important role in increasing confidence in the structural basis of computational predictions. Post-MD analyses such as Principal Component Analysis (PCA), pharmacophore-based conformational clustering, or pocket-volume tracking (POVME) were not included in this study. These tools provide an enhanced understanding of the conformational dynamics and binding site plasticity and will be explored in future studies. Also, the virtual screening pipeline was not benchmarked using enrichment metrics such as ROC-AUC or EF1%, nor was a decoy set employed for validation. These steps are critical for assessing the robustness of hit identification and will be incorporated in subsequent retrospective validation studies. While STAT3 reporter assays provide evidence of functional inhibition, they offer limited insight into specific molecular interactions. Future studies will be focused on direct binding studies using experimental techniques such as surface plasmon resonance (SPR), cellular thermal shift assays (CETSA), along with mechanistic insights into key interaction sites from QM/MM calculations. These higher-order validations are beyond the scope of the present study but are planned as part of an extended study to refine and validate the proposed inhibitors. These approaches will allow for deeper mechanistic insights, binding confirmation, and lead optimization, and deepen the translational value of our findings in subsequent phases of the work. 

## 4. Conclusions

Interleukin-6 is a pleiotropic, pro inflammatory cytokine produced by a variety of cell types, including lymphocytes, monocytes, and fibroblasts. hIL-6 is prominent in the etiology of several diseases and the cytokine storm. Anti-IL-6 strategies [90,91] may be broadly applicable to a wide range of medical conditions, such as pericarditis, gout, type 2 diabetes, and others that are still under investigation. However, hIL-6 also plays an important role in immune responses and tissue regeneration. Therefore, hIL-6 has a narrow therapeutic window, and long-term inhibition can result in numerous adverse effects, such as increased vulnerability to bacterial and fungal infections. To overcome these limitations, it will be important to develop a variety of drugs that address the specific functions of hIL-6. Although therapeutic antibodies against hIL-6 have been developed, small-molecule therapeutics, which have the clear advantage of oral availability, are lacking.

Here, a structure-based target site prediction approach was used, informed by understanding the detailed mechanism of formation of the IL-6/IL-6Rα complex that is pertinent in triggering the signaling cascade of hIL-6. Structural information on the contact sites of hIL-6 involved in the formation of the binary complex was employed in virtual high-throughput screening through targeted ensemble docking studies. The top 20 scoring ligands from enamine, as shown in Table 1, as well as the three other NCI compounds as shown in Table 2 from the docking studies, were subjected to in vitro analysis. The ligand with the second highest binding affinity, as shown in Table 1 from in silico studies, showed an ~84% inhibitory effect on hIL6 binding for the in vitro experimental assay and an IC_50_ value of 2.7 ± 0.5 µM.

Cell assays are excellent for identifying compounds that produce a desired phenotype, but do not confirm the proposed mechanism of action or provide details of specific target binding. For the perturbation of protein–protein interactions, obtaining this type of information is non-trivial as it would be of use in lead optimization and target validation studies. Pharmacokinetic profiling, including membrane permeability assessment, will also be essential for lead compound validation. 

Building on the predicted interaction profile of Z169667518, future lead optimization efforts may focus on introducing polar substituents at solvent-exposed regions to improve solubility while preserving key interactions. The drug development pathway would further involve analysis of structure–activity relationships, free energy perturbation (FEP)-guided analog design, and in silico screening of ADME and toxicity profiles. Promising analogs would then be synthesized and evaluated through in vitro assays, followed by in vivo studies to assess pharmacokinetics, efficacy, and safety in relevant disease models. Broader cell-based screening would be used to rule out off-target effects and prioritize lead candidates for further development. 

We acknowledge that further studies, including detailed binding assays and structural analysis, will be valuable in fully characterizing the interaction between the small molecule and the IL-6 receptor complex. Notwithstanding, the discovery of this ligand demonstrates that biophysical structure-based computational methods can be combined with assays to find small molecules capable of preventing the formation of the hIL-6/IL-6Rα complex to inhibit hIL6-induced STAT3 reporter activities. 

## Figures and Tables

**Table 2:** Drug likeness properties [87] of 3 NCI ligands with the highest docking scores subjected to in vitro studies.

Compound ID	Mol. Formula	Mol. Wt.	logP	Net Charge	H-Bond Donors	H-Bond Acceptors	tPSA	Docking Score (kcal/mol)
NSC39921	C_43_H_43_N_3_O_6_S_2_	762.0	6.3	0	2	8	192.9	−11.1
NSC13989	C_34_H_18_O_2_	458.5	8.8	0	0	2	34.1	−10.6
NSC171279	C_30_H_30_	390.6	7.1	0	0	0	0	−10.6

## Data Availability

The data will be made available upon request to the authors.

## References

[1] Ehlers M., Grötzinger J., Dehon F.D., Müllberg J., Brakenhoff J.P., Liu J., Wollmer A., Rose-John S. (1994). Identification of Two Novel Regions of Human IL-6 Responsible for Receptor Binding and Signal Transduction. J. Immunol..

[2] Taga T., Kishimoto T. (1997). Gp130 and the Interleukin-6 Family of Cytokines. Annu. Rev. Immunol..

[3] Kishimoto T., Akira S., Narazaki M., Taga T. (1995). Interleukin-6 Family of Cytokines and Gp130. Blood.

[4] Heinrich P.C., Behrmann I., Müller-Newen G., Schaper F., Graeve L. (1998). Interleukin-6-Type Cytokine Signalling through the Gp130/Jak/STAT Pathway. Biochem. J..

[5] Scheller J., Chalaris A., Schmidt-Arras D., Rose-John S. (2011). The Pro-and Anti-Inflammatory Properties of the Cytokine Interleukin-6. Biochim. Biophys. Acta (BBA) Mol. Cell Res..

[6] Becker C., Fantini M.C., Schramm C., Lehr H.A., Wirtz S., Nikolaev A., Burg J., Strand S., Kiesslich R., Huber S. (2004). TGF-Suppresses Tumor Progression in Colon Cancer by Inhibition of IL-6 trans-Signaling. Immunity.

[7] Grivennikov S., Karin E., Terzic J., Mucida D., Yu G.-Y., Vallabhapurapu S., Scheller J., Rose-John S., Cheroutre H., Eckmann L. (2009). IL-6 and Stat3 Are Required for Survival of Intestinal Epithelial Cells and Development of Colitis-Associated Cancer. Cancer Cell.

[8] Tamura T., Udagawa N., Takahashi N., Miyaura C., Tanaka S., Yamada Y., Koishihara Y., Ohsugi Y., Kumaki K., Taga T. (1993). Soluble Interleukin-6 Receptor Triggers Osteoclast Formation by Interleukin 6. Proc. Natl. Acad. Sci. USA.

[9] Hénaut L., Massy Z.A. (2018). New Insights into the Key Role of Interleukin 6 in Vascular Calcification of Chronic Kidney Disease. Nephrol. Dial. Transplant..

[10] Hirano T., Matsuda T., Turner M., Miyasaka N., Buchan G., Tang B., Sato K., Shimi M., Maid R., Feldmann M. (1988). Excessive Production of Interleukin 6/B Cell Stimulatory Factor-2 in Rheumatoid Arthritis. Eur. J. Immunol..

[11] McGonagle D., Sharif K., O’REgan A., Bridgewood C. (2020). The Role of Cytokines Including Interleukin-6 in COVID-19 Induced Pneumonia and Macrophage Activation Syndrome-Like Disease. Autoimmun. Rev..

[12] McElvaney O.J., Curley G.F., Rose-John S., McElvaney N.G. (2021). Interleukin-6: Obstacles to Targeting a Complex Cytokine in Critical Illness. Lancet Respir. Med..

[13] Egorov A., Chasnyk V., Kostik M., Snegireva L., Kalashnikova O., Dubko M., Masalova V., Likhacheva T., Fedorova E. (2014). Anemia in Children with JIA: Is It Really Driven by Hepcidin Level, or by a Set of Factors of a Chronic Disease. Pediatr. Rheumatol..

[14] Nishimoto N., Murakami M., Tomiita M. (2012). Tomiita Tocilizumab in the Treatment of Systemic Juvenile Idiopathic Arthritis. Open Access Rheumatol. Res. Rev..

[15] Emery P., Keystone E., Tony H.P., Cantagrel A., Van Vollenhoven R., Sanchez A., Alecock E., Lee J., Kremer J. (2008). IL-6 Receptor Inhibition with Tocilizumab Improves Treatment Outcomes in Patients with Rheumatoid Arthritis Refractory to Anti-Tumour Necrosis Factor Biologicals: Results from a 24-Week Multicentre Randomised Placebo-Controlled Trial. Ann. Rheum. Dis..

[16] Jordan S.C., Choi J., Kim I., Wu G., Toyoda M., Shin B., Vo A. (2017). Interleukin-6, A Cytokine Critical to Mediation of Inflammation, Autoimmunity and Allograft Rejection. Transplantation.

[17] Chakraborty C., Sharma A.R., Bhattacharya M., Sharma G., Lee S., Agoramoorthy G. (2020). COVID-19: Consider IL-6 Receptor Antagonist for the Therapy of Cytokine Storm Syndrome in SARS-CoV-2 Infected Patients. J. Med. Virol..

[18] Casper C., Chaturvedi S., Munshi N., Wong R., Qi M., Schaffer M., Bandekar R., Hall B., Van de Velde H., Vermeulen J. (2015). Analysis of Inflammatory and Anemia-Related Biomarkers in a Randomized, Double-Blind, Placebo-Controlled Study of Siltuximab (Anti-IL6 Monoclonal Antibody) in Patients With Multicentric Castleman Disease. Clin. Cancer Res..

[19] Wang C.-Y., Babitt J.L. (2016). Hepcidin Regulation in the Anemia of Inflammation. Curr. Opin. Hematol..

[20] Ancrile B., Lim K.-H., Counter C.M. (2007). Oncogenic Ras-Induced Secretion of IL6 Is Required for Tumorigenesis. Genes Dev..

[21] Zhang S., Chen B., Wang B., Chen H., Li Y., Cao Q., Zhong J., Shieh M.-J., Ran Z., Tang T. (2023). Effect of Induction Therapy With Olamkicept vs Placebo on Clinical Response in Patients With Active Ulcerative Colitis. JAMA.

[22] Waetzig G.H., Chalaris A., Rosenstiel P., Suthaus J., Holland C., Karl N., Uriarte L.V., Till A., Scheller J., Grötzinger J. (2010). N-Linked Glycosylation Is Essential for the Stability but Not the Signaling Function of the Interleukin-6 Signal Transducer Glycoprotein 130. J. Biol. Chem..

[23] Tenhumberg S., Waetzig G.H., Chalaris A., Rabe B., Seegert D., Scheller J., Rose-John S., Grötzinger J. (2008). Structure-guided Optimization of the Interleukin-6 Trans-signaling Antagonist sgp130. J. Biol. Chem..

[24] Rose-John S. (2003). Interleukin-6 Biology Is Coordinated by Membrane Bound and Soluble Receptors. Acta Biochim. Pol..

[25] Garbers C., Thaiss W., Jones G.W., Waetzig G.H., Lorenzen I., Guilhot F., Lissilaa R., Ferlin W.G., Grötzinger J., Jones S.A. (2011). Inhibition of Classic Signaling Is a Novel Function of Soluble Glycoprotein 130 (sgp130), Which Is Controlled by the Ratio of Interleukin 6 and Soluble Interleukin 6 Receptor. J. Biol. Chem..

[26] Jostock T., Müllberg J., Özbek S., Atreya R., Blinn G., Voltz N., Fischer M., Neurath M.F., Rose-John S. (2001). Soluble Gp130 Is the Natural Inhibitor of Soluble Interleukin-6 Receptor Transsignaling Responses. Eur. J. Biochem..

[27] Van Vollenhoven R.F., Fleischmann R., Cohen S., Lee E.B., Meijide J.A.G., Wagner S., Forejtova S., Zwillich S.H., Gruben D., Koncz T. (2012). Tofacitinib or Adalimumab versus Placebo in Rheumatoid Arthritis. N. Engl. J. Med..

[28] Genovese M.C., Smolen J.S., Weinblatt M.E., Burmester G.R., Meerwein S., Camp H.S., Wang L., Othman A.A., Khan N., Pangan A.L. (2016). Efficacy and Safety of ABT-494, a Selective JAK-1 Inhibitor, in a Phase IIb Study in Patients With Rheumatoid Arthritis and an Inadequate Response to Methotrexate. Arthritis Rheumatol..

[29] Kremer J.M., Emery P., Camp H.S., Friedman A., Wang L., Othman A.A., Khan N., Pangan A.L., Jungerwirth S., Keystone E.C. (2016). A Phase IIb Study of ABT–494, a Selective JAK–1 Inhibitor, in Patients With Rheumatoid Arthritis and an Inadequate Response to Anti–Tumor Necrosis Factor Therapy. Arthritis Rheumatol..

[30] Rubbert-Roth A., Enejosa J., Pangan A.L., Haraoui B., Rischmueller M., Khan N., Zhang Y., Martin N., Xavier R.M. (2020). Trial of Upadacitinib or Abatacept in Rheumatoid Arthritis. N. Engl. J. Med..

[31] Hong S.-S., Choi J.H., Lee S.Y., Park Y.-H., Park K.-Y., Lee J.Y., Kim J., Gajulapati V., Goo J.-I., Singh S. (2015). A Novel Small-Molecule Inhibitor Targeting the IL-6 Receptor Subunit, Glycoprotein 130. J. Immunol..

[32] Swaroop A.K., Namboori P.K.K., Esakkimuthukumar M., Praveen T.K., Nagarjuna P., Patnaik S.K., Selvaraj J. (2023). Leveraging Decagonal In-Silico Strategies for Uncovering IL-6 Inhibitors with Precision. Comput. Biol. Med..

[33] Schultz D.C., Pan L., Wang T., Booker C., Hyder I., Hanold L., Rubin G., Ding Y., Lin J., Li C. (2023). Carbohydrate-Small Molecule Hybrids as Lead Compounds Targeting IL-6 Signaling. Molecules.

[34] Zinzalla G., Haque M.R., Basu B.P., Anderson J., Kaye S.L., Haider S., Hasan F., Antonow D., Essex S., Rahman K.M. (2010). A Novel Small-Molecule Inhibitor of IL-6 Signalling. Bioorganic Med. Chem. Lett..

[35] Chen Q., Niu X., Li N. (2018). Exploring the Natural Chemiome to Target Interleukin-6 Receptor (IL-6R) Cytokines: An Atomic Scale Investigation for Novel Rheumatoid Arthritis Drug Discovery. Braz. J. Pharm. Sci..

[36] Yoshizaki K., Matsuda T., Nishimoto N., Kuritani T., Taeho L., Aozasa K., Nakahata T., Kawai H., Tagoh H., Komori T. (1989). Pathogenic Significance of Interleukin-6 (IL-6/BSF-2) in Castleman’s Disease. Blood.

[37] Villar-Fincheira P., Sanhueza-Olivares F., Norambuena-Soto I., Cancino-Arenas N., Hernandez-Vargas F., Troncoso R., Gabrielli L., Chiong M. (2021). Role of Interleukin-6 in Vascular Health and Disease. Front. Mol. Biosci..

[38] Hirano T. (2020). IL-6 in Inflammation, Autoimmunity and Cancer. Int. Immunol..

[39] Shun T.Y., Lazo J.S., Sharlow E.R., Johnston P.A. (2011). Identifying Actives from HTS Data Sets: Practical Approaches for the Selection of an Appropriate HTS Data-Processing Method and Quality Control Review. Slas Discov. Adv. Sci. Drug Discov..

[40] Gupta M., Ha K., Agarwal R., Quarles L.D., Smith J.C. (2020). Molecular dynamics analysis of the binding of human interleukin-6 with interleukin-6-receptor. Proteins Struct. Funct. Bioinform..

[41] Wang J., Qiao C., Xiao H., Lin Z., Li Y., Zhang J., Shen B., Fu T., Feng J. (2016). Structure-Based Virtual Screening and Characterization of a Novel IL-6 Antagonistic Compound from Synthetic Compound Database. Drug Des. Dev. Ther..

[42] Tran Q.-H., Nguyen Q.-T., Vo N.-Q.-H., Mai T.T., Tran T.-T.-N., Tran T.-D., Le M.-T., Trinh D.-T.T., Thai K.-M. (2022). Structure-Based 3D-Pharmacophore Modeling to Discover Novel Interleukin 6 Inhibitors: An in Silico Screening, Molecular Dynamics Simulations and Binding Free Energy Calculations. PLoS ONE.

[43] Nada H., Sivaraman A., Lu Q., Min K., Kim S., Goo J.-I., Choi Y., Lee K. (2023). Perspective for Discovery of Small Molecule IL–6 Inhibitors through Study of Structure–Activity Relationships and Molecular Docking. J. Med. Chem..

[44] Falcon W.E., Ellingson S.R., Smith J.C., Baudry J.Y. (2019). Ensemble Docking in Drug Discovery: How Many Protein Configurations from Molecular Dynamics Simulations are Needed To Reproduce Known Ligand Binding?. J. Phys. Chem. B.

[45] Amaro R.E., Baudry J., Chodera J., Demir Z., McCammon J.A., Miao Y., Smith J.C. (2018). Ensemble Docking in Drug Discovery. Biophys. J..

[46] Acharya A., Agarwal R., Baker M.B., Baudry J., Bhowmik D., Boehm S., Byler K.G., Chen S.Y., Coates L., Cooper C.J. (2020). Supercomputer-Based Ensemble Docking Drug Discovery Pipeline with Application to COVID-19. J. Chem. Inf. Model..

[47] Agarwal R., Bensing B.A., Mi D., Vinson P.N., Baudry J., Iverson T.M., Smith J.C. (2020). Structure Based Virtual Screening Identifies Small Molecule Effectors for the Sialoglycan Binding Protein Hsa. Biochem. J..

[48] Davis C.C., Shah K.S., Lechowicz M.J. (2015). Clinical Development of Siltuximab. Curr. Oncol. Rep..

[49] Somers W., Stahl M., Seehra J.S. (1997). 9 Acrystal structure of interleukin 6: Implications for a novel mode of receptor dimerization and signaling. EMBO J..

[50] Boulanger M.J., Chow D.-C., Brevnova E.E., Garcia K.C. (2003). Hexameric Structure and Assembly of the Interleukin-6/IL-6-Receptor/gp130 Complex. Science.

[51] Song Y., DiMaio F., Wang R.Y.-R., Kim D., Miles C., Brunette T., Thompson J., Baker D. (2013). High-Resolution Comparative Modeling with RosettaCM. Structure.

[52] Jorgensen W.L., Chandrasekhar J., Madura J.D., Impey R.W., Klein M.L. (1983). Comparison of Simple Potential Functions for Simulating Liquid Water. J. Chem. Phys..

[53] Jo S., Kim T., Iyer V.G., Im W. (2008). CHARMM-GUI: A Web-Based Graphical User Interface for CHARMM. J. Comput. Chem..

[54] Phillips J.C., Braun R., Wang W., Gumbart J., Tajkhorshid E., Villa E., Chipot C., Skeel R.D., Kalé L., Schulten K. (2005). Scalable Molecular Dynamics with NAMD. J. Comput. Chem..

[55] Huang J., MacKerell A.D. (2013). CHARMM36 All-Atom Additive Protein Force Field: Validation Based on Comparison to NMR Data. J. Comput. Chem..

[56] Feller S.E., Zhang Y., Pastor R.W., Brooks B.R. (1995). Constant Pressure Molecular Dynamics Simulation: The Langevin Piston Method. J. Chem. Phys..

[57] Darden T., York D., Pedersen L. (1993). Particle mesh Ewald: An Nlog(N) method for Ewald sums in large systems. J. Chem. Phys..

[58] Ryckaert J.-P., Ciccotti G., Berendsen H.J.C. (1977). Numerical Integration of the Cartesian Equations of Motion of a System with Constraints: Molecular Dynamics of n-Alkanes. J. Comput. Phys..

[59] Darnell S.J., Page D., Mitchell J.C. (2007). An Automated Decision-Tree Approach to Predicting Protein Interaction Hot Spots. Proteins: Struct. Funct. Bioinform..

[60] Zhu X., Mitchell J.C. (2011). KFC2: A Knowledge-Based Hot Spot Prediction Method Based on Interface Solvation, Atomic Density, and Plasticity Features. Proteins: Struct. Funct. Bioinform..

[61] Leebeek F.W., Kariya K., Schwabe M., Fowlkes D.M. (1992). Identification of a Receptor Binding Site in the Carboxyl Terminus of Human Interleukin-6. J. Biol. Chem..

[62] Fontaine V., Savino R., Arcone R., De Wit L., Brakenhoff J.P.J., Content J., Ciliberto G. (1993). Involvement of the Arg179 in the Active Site of Human IL-6. Eur. J. Biochem..

[63] Lütticken C., Krüttgen A., Möller C., Heinrich P.C., Rose-John S. (1991). Evidence for the importance of a positive charge and an &alpha;-helical structure of the C-terminus for biological activity of human IL-6. FEBS Lett..

[64] Krüttgen A., Rose-John S., Möller C., Wroblowski B., Wollmer A., Müllberg J., Hirano T., Kishimoto T., Heinrich P.C. (1990). Structure-Function Analysis of Human Interleukin-6. FEBS Lett..

[65] Abraham M.J., Murtola T., Schulz R., Páll S., Smith J.C., Hess B., Lindahl E. (2015). GROMACS: High Performance Molecular Simulations through Multi-Level Parallelism from Laptops to Supercomputers. SoftwareX.

[66] Ellingson S.R., Smith J.C., Baudry J. (2013). VinaMPI: Facilitating Multiple Receptor High-Throughput Virtual Docking on High-Performance Computers. J. Comput. Chem..

[67] Voigt J.H., Bienfait B., Wang S., Nicklaus M.C. (2001). Comparison of the NCI Open Database with Seven Large Chemical Structural Databases. J. Chem. Inf. Comput. Sci..

[68] Enamine Discovery Diversity Set. https://enamine.net/compound-libraries/diversity-libraries/dds-50240.

[69] Enamine PPI Library. https://enamine.net/compound-libraries/targeted-libraries/ppi-library.

[70] Novick P.A., Ortiz O.F., Poelman J., Abdulhay A.Y., Pande V.S. (2013). SWEETLEAD: An In Silico Database of Approved Drugs, Regulated Chemicals, and Herbal Isolates for Computer-Aided Drug Discovery. PLoS ONE.

[71] Sanner M.F. (1999). Python: A Programming Language for Software Integration and Development. J. Mol. Graph. Model..

[72] O’BOyle N.M., Banck M., James C.A., Morley C., Vandermeersch T., Hutchison G.R. (2011). Open Babel: An Open Chemical Toolbox. J. Cheminform..

[73] Lagorce D., Bouslama L., Becot J., Miteva M.A., Villoutreix B.O. (2017). FAF-Drugs4: Free ADME-Tox Filtering Computations for Chemical Biology and Early Stages Drug Discovery. Bioinformatics.

[74] Rogers D.M., Agarwal R., Vermaas J.V., Smith M.D., Rajeshwar R.T., Cooper C., Sedova A., Boehm S., Baker M., Glaser J. (2023). SARS-CoV2 Billion-Compound Docking. Sci. Data.

[75] Lipinski C.A. (2000). Drug-like Properties and the Causes of Poor Solubility and Poor Permeability. J. Pharmacol. Toxicol. Methods.

[76] Lipinski C.A., Lombardo F., Dominy B.W., Feeney P.J. (1997). Experimental and Computational Approaches to Estimate Solubility and Permeability in Drug Discovery and Development Settings. Adv. Drug Deliv. Rev..

[77] Leeson P.D., Bento A.P., Gaulton A., Hersey A., Manners E.J., Radoux C.J., Leach A.R. (2021). Target-Based Evaluation of Drug-Like Properties and Ligand Efficiencies. J. Med. Chem..

[78] Lipinski C.A. (2004). Lead-and Drug-like Compounds: The Rule-of-Five Revolution. Drug Discov. Today Technol..

[79] Doak B.C., Kihlberg J. (2016). Drug discovery beyond the rule of 5—Opportunities and challenges. Expert Opin. Drug Discov..

[80] Oprea T.I. (2000). Property distribution of drug-related chemical databases*. J. Comput. Mol. Des..

[81] Verboogen D.R.J., Revelo N.H., Beest M.T., Van Den Bogaart G. (2018). Interleukin-6 Secretion Is Limited by Self-Signaling in Endosomes. J. Mol. Cell Biol..

[82] Barillé S., Bataille R., Amiot M. (2000). The Role of Interleukin-6 and Interleukin-6/Interleukin-6 Receptor-Alpha Complex in the Pathogenesis of Multiple Myeloma. Eur. Cytokine Netw..

[83] Sun Y., Vandenbriele C., Kauskot A., Verhamme P., Hoylaerts M.F., Wright G.J. (2015). A Human Platelet Receptor Protein Microarray Identifies FcepsilonR1alpha as an Activating PEAR1 Ligand. Mol. Cell Proteomics.

[84] Baeg G.-H., Zhou R., Perrimon N. (2005). Genome-Wide RNAi Analysis of JAK/STAT Signaling Components in Drosophila. Genes Dev..

[85] Xiao Z., Liu J., Liu S.-H., Petridis L., Cai C., Cao L., Wang G., Chin A.L., Cleveland J.W., Ikedionwu M.O. (2022). Novel Small Molecule Fibroblast Growth Factor 23 Inhibitors Increase Serum Phosphate and Improve Skeletal Abnormalities in Hyp Mice. Mol. Pharmacol..

[86] Horowitz S., Trievel R.C. (2012). Carbon-Oxygen Hydrogen Bonding in Biological Structure and Function. J. Biol. Chem..

[87] Irwin J.J., Tang K.G., Young J., Dandarchuluun C., Wong B.R., Khurelbaatar M., Moroz Y.S., Mayfield J., Sayle R.A. (2020). ZINC20A Free Ultralarge-Scale Chemical Database for Ligand Discovery. J. Chem. Inf. Model..

[88] Zia K., Nur-E-Alam M., Ahmad A., Ul-Haq Z. (2024). Taming the cytokine storm: Small molecule inhibitors targeting IL-6/IL-6 receptor. Mol. Divers..

[89] Tran Q.-H., Nguyen Q.-T., Tran T.-T.N., Tran T.-D., Le M.-T., Trinh D.-T.T., Tran V.-T., Tran V.-H., Thai K.-M. (2022). Identification of Small Molecules as Potential Inhibitors of Interleukin 6: A Multi-Computational Investigation. Mol. Divers..

[90] Kapriniotis K., Lampridis S., Mitsos S., Patrini D., Lawrence D.R., Panagiotopoulos N. (2018). Biologic Agents in the Treatment of Multicentric Castleman Disease. Turk. Thorac. J..

[91] Imazio M., Lazaros G., Gattorno M., LeWinter M., Abbate A., Brucato A., Klein A. (2021). Anti-Interleukin-1 Agents for Pericarditis: A Primer for Cardiologists. Eur. Heart J..

